# Supplementary material for: Altered microRNA Transcriptome in Cultured Human Liver Cells upon Infection with Ebola Virus
Source: Int J Mol Sci. 2021 Apr 6;22(7):3792. doi: 10.3390/ijms22073792 (PMC8038836; doi:10.3390/ijms22073792)
Supplement: Supplementary file 1 [file ijms-22-03792-s001.zip › Supplementary_File/D_ KEGG_Analysis_Results/16-30nt_pathway_Mayinga-96h-Huh7_vs_Control-96h-Huh7_down.mature_mirna_targets/hsa_EnrichmentScoreDotPlot.pdf]

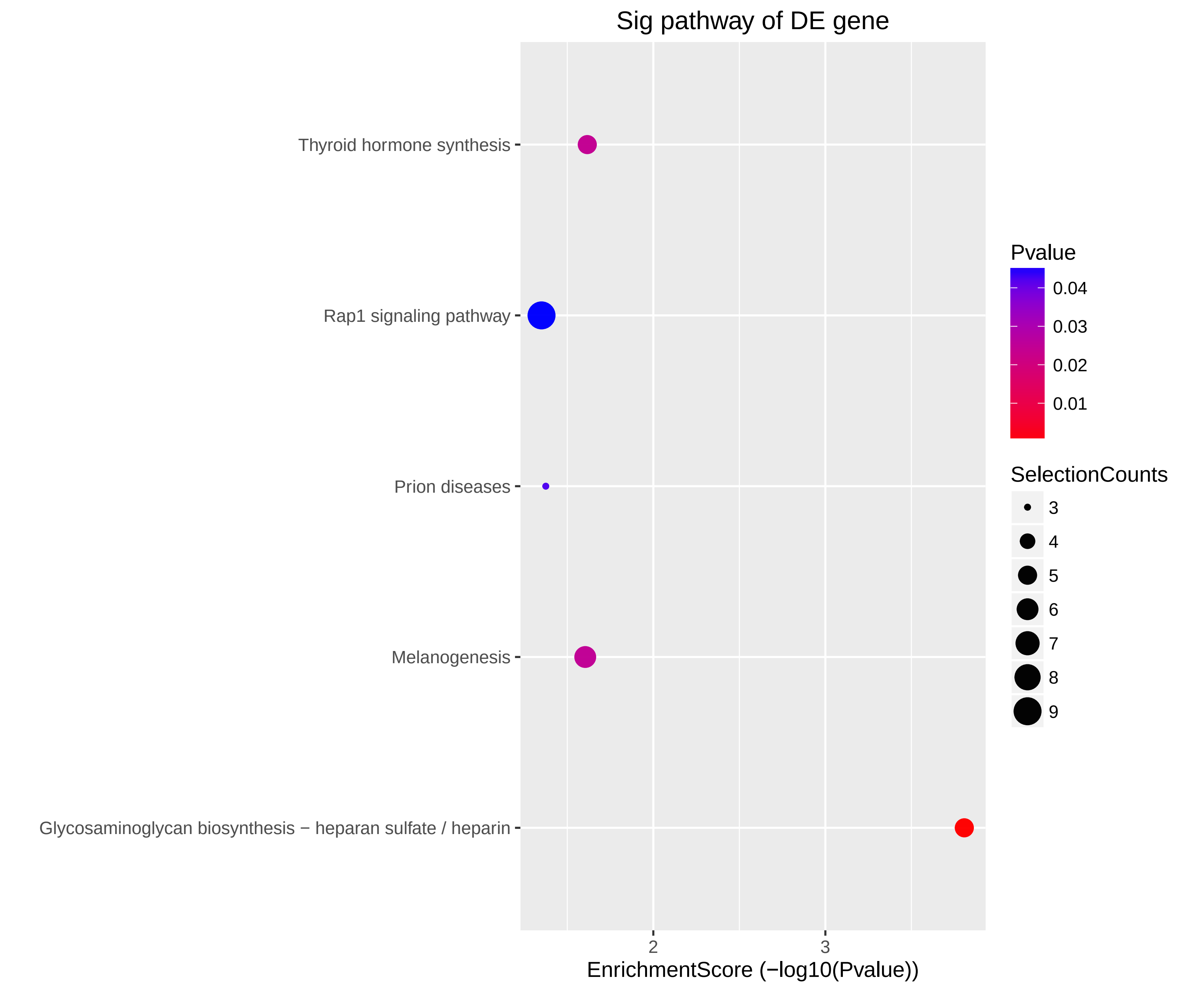
Sig pathway of DE gene
Thyroid hormone synthesis
Rap1 signaling pathway
Prion diseases
Melanogenesis
Glycosaminoglycan biosynthesis − heparan sulfate / heparin
2
3
EnrichmentScore (−log10(Pvalue))
Pvalue
0.04
0.03
0.02
0.01
SelectionCounts
3
4
5
6
7
8
9
